# Role of Endoscopic Ultrasound in Staging and Vascular Assessment of Pancreatic Cancer

**DOI:** 10.7759/cureus.53988

**Published:** 2024-02-10

**Authors:** Mohammed A El-Nady, Khalid A Ead, Mustafa A Haridy, Nour Shaheen, Abdulqadir J Nashwan, Saad R Abdelwahid, Mohammed F Mohammed, Omran Mohamed, Safwat S Sawy, Emad Abdelrazzak, Amro M Hassan

**Affiliations:** 1 Medicine, Cairo University, Cairo, EGY; 2 Hepatology, Gastroenterology, and Infectious Diseases, Al-Azhar University, Assiut, EGY; 3 Medicine, Alexandria University, Alexandria, EGY; 4 Nursing, Hamad Medical Corporation, Doha, QAT; 5 Radiodiagnosis, Al-Azhar University, Assiut, EGY

**Keywords:** contrast-enhanced computed tomography (cect), ct abdomen, vascular analysis, pancreatic cancer, vascular invasion, eus

## Abstract

Introduction

Computed tomography (CT) of the abdomen with contrast stands as the gold standard for assessing pancreatic cancer, encompassing both staging and vascular analysis. However, not all patients are suitable candidates for contrast-enhanced CT (CECT) scans due to factors such as contrast agent allergies, pregnancy, renal impairment, radiation risks, and limited tissue sampling capability in CECT scans of the abdomen. In light of these challenges, this study evaluated the diagnostic capabilities of endoscopic ultrasound (EUS) compared to CECT for staging and vascular assessment of pancreatic cancer.

Methods

Fifty patients diagnosed with pancreatic cancer underwent evaluations using both CECT scans and EUS, focusing on staging and vascular invasion assessment. Vascular evaluation was carried out using a categorization system based on EUS findings, classifying them into three types based on the tumor-vessel relationship: Type 1 indicating clear invasion or encasement of a vessel by a tumor or contact with a vessel wall exceeding 180 degrees, Type 2 representing abutment, wherein a tumor contacts a vessel wall but at an angle less than 180 degrees, and Type 3 implying clear non-invasion, where a discernible distance exists between a tumor and a vessel. In this categorization, Type 1 and Type 2 indicated signs of vascular invasion, while Type 3 indicated vascular non-invasion. These findings were subsequently compared to the results from CECT scans. The endoscopist performing EUS was blinded to the CT outcomes prior to the examination.

Results

Regarding pancreatic cancer staging, EUS exhibited remarkable sensitivity, specificity, and accuracy rates of 100% according to the T criterion.As for vascular invasion assessment, EUS demonstrated sensitivity, specificity, and accuracy of 100%, 95.93%, and 96%, respectively, for venous invasion. For arterial invasion, the figures were 95.65% sensitivity, 100% specificity, and an overall accuracy of 99.5%.

Conclusion

EUS is an effective modality for evaluating both staging and vascular invasion in pancreatic cancer, boasting exceptional sensitivity, specificity, and accuracy rates. The findings are robust enough to consider EUS a viable alternative to CT scans in evaluation, with the added advantage of EUS offering tissue sampling capability.

## Introduction

Pancreatic cancer stands as a malignancy characterized by a dismal prognosis, boasting a mere five-year survival rate that scarcely surpasses 10% [1]. Globally, it ranks as the sixth most prevalent cancer and constitutes the third principal cause of cancer-induced mortality in the United States, further attesting to its profound impact [2]. The potential for curative intervention lies primarily in surgical resection with sufficient safety margins when metastatic lesions are absent. Regrettably, the diagnosis of this malignancy frequently transpires in advanced stages, thereby rendering surgical intervention feasible only in a confined subset of cases [3].

The decision to proceed with surgical resection of pancreatic cancer pivots not solely on tumor dimensions but substantially on the presence of metastases and the extent of invasion into contiguous major vessels, which encompass a range including the common hepatic artery (CHA), superior mesenteric artery (SMA), superior mesenteric vein (SMV), portal vein (PV), celiac artery (CA), splenic artery (SPA), and splenic vein (SPV) [4]. Although CT with intravenous contrast administration currently reigns as the gold standard for the initial staging of pancreatic cancer, certain patients are precluded from undergoing contrast-enhanced abdominal CT examinations due to contraindications like contrast agent allergies, compromised renal function, and pregnancy [5].

Endoscopic ultrasonography (EUS), a form of endoscopic imaging, affords unparalleled resolution and intricate depiction of the pancreas when contrasted with conventional CT scans. Integrating an ultrasound transducer onto the endoscope, EUS generates meticulous and unhindered imagery devoid of nephrotoxic contrast exposure [6]. Notably, EUS is pivotal in diagnostic tissue sampling (fine needle aspiration or fine needle biopsy) and the regional staging of pancreatic cancer. The progressive refinement of EUS methodologies has amplified its significance as an indispensable instrument for the comprehensive management, staging, and vascular scrutiny of pancreatic cancer [7-9]. However, consensus concerning the precise role of EUS in the primary evaluation of pancreatic cancer remains elusive [5].

Henceforth, the present study has been conceived with the specific objective of assessing the diagnostic efficacy of EUS in the context of pancreatic cancer staging and the discernment of significant vascular invasion. A comparative analysis with contrast-enhanced abdomen CT shall be undertaken to elucidate the potency of EUS in these critical dimensions.

This article was previously posted to the Research Square preprint server on December 29th, 2022.

## Materials and methods

Study design

This investigation adopts a cross-sectional design to assess the diagnostic prowess of endoscopic ultrasound (EUS) in pancreatic cancer staging and vascular evaluation, juxtaposed against contrast-enhanced computed tomography (CECT).

Setting and duration

Conducted at the Al-Ibrashi Endoscopy Unit within the Internal Medicine Hospital of Cairo University, this study spanned from January 2020 to October 2022, with an exclusion period from March 2020 to September 2020 due to the facility's temporary transformation into an isolation center for COVID-19 patients.

Participants

Out of 89 patients identified with pancreatic focal lesions through abdominal ultrasound, 50 individuals were selected based on subsequent histopathological confirmation of pancreatic cancer by fine-needle aspiration biopsy (FNAB). Inclusion criteria entailed a minimum age of 18 years and a confirmed diagnosis of pancreatic cancer via FNAB. Exclusion criteria encompassed age below 18 years, previous gastric surgery, pregnancy or lactation, prior radiation therapy or chemotherapy, contraindications to CECT, including chronic renal failure or contrast material allergy, and negative FNAB results for malignancy.

Workup

Eligible subjects underwent comprehensive evaluation, which encompassed: (1) thorough medical history and clinical examination; (2) laboratory investigations following overnight fasting, including complete blood count, liver function tests (alanine transaminase (ALT), aspartate transaminase (AST), international normalizing ratio (INR), serum bilirubin), renal function tests (urea, creatinine), and tumor markers such as CEA and CA19.9; (3) CECT of the abdomen and pelvis for locoregional staging and vascular appraisal, employing a pancreas CT protocol involving triphasic imaging with thin slices using a 16-channel multi-detector CT scanner; (4) EUS examination under general anesthesia utilizing a 7.5 MHz echoendoscope (Pentax FG 38UX or Hitachi ultrasound EUB 525). Based on needle availability, FNAB from the pancreatic focal lesion was performed using echo tip needles of either 22 gauge or 19 gauge. Locoregional staging adhered to the general guidelines of the American Joint Committee on Cancer 8th edition. Vascular evaluation was conducted in accordance with the tumor-vessel relationship, stratified into three categories: Type I for clear invasion or encasement, Type II for abutment, and Type III for non-invasion. Types I and II indicated vascular invasion, whereas Type III indicated non-invasion. The specific vessels evaluated depended on the tumor's location.

Statistical analysis

Data collected were subjected to meticulous revision, organization, tabulation, and statistical analysis through IBM SPSS Statistics (Version 26). Descriptive statistics were employed for categorical and continuous variables, and various statistical tests (e.g., Student's t-test, Fisher's exact test) were utilized for comparisons. Sensitivity, specificity, negative predictive value (NPV), positive predictive value (PPV), and accuracy of EUS in diagnosing vascular invasion were computed using Excel.

## Results

Demographic characteristics and presenting symptoms

In this cross-sectional study, the mean age of the enrolled patients was 62.32 ± 7.82 years, with 72% (n=36) male and 28% (n=14) female. Among the study participants, 64% (n=32) were smokers. The most common presenting symptom was jaundice, which was observed in 66% (n=33) of patients, followed by abdominal pain in 22% (n=11), incidental discovery in 10% (n=5), and abdominal swelling in 2% (n=1) as shown in Table 1 and Table 2.

**Table 1:** Demographic characteristics of the studied patients

Parameters	All cases, N=50	
Age (years), mean + SD	62.32±7.82	
Gender	Frequency (n)	Percentage (%)
Male	36	72%
Female	14	28%
Smoking	N	%
Non-smoker	18	36%
Smoker	32	64%

**Table 2:** The main complaint of the studied patients (n=50)

Main complaint	Frequency (n)	Percentage (%)
Jaundice	33	66.0%
Abdominal pain	11	22.0%
Accidently discovered	5	10.0%
Abdominal swelling	1	2.0%
Total	50	100.0%

Laboratory investigations

Laboratory data of the studied patients are shown in Table 3.

**Table 3:** Laboratory data of the studied patients (n=50) Laboratory investigation of the studied patients presented as mean ± SD and median and range. AST, aspartate transaminase; INR, international normalizing ratio; RBG, random blood glucose; HB, hemoglobin; RBC, red blood corpuscle; MCV, mean corpuscular volume; PLT, platelets; WBC, white blood cells; CEA, carcinoembryonic antigen; CA19.9, carbohydrate antigen 19.9

Parameters	Mean + SD		Range	
	Mean	SD	Minimum	Maximum
ALT (U/L)	69.31	66.78	12	276
AST (U/L)	66.78	62.4	12	267
Total bilirubin (mg/dL)	8.74	7.82	0.41	26.5
Direct bilirubin (mg/dL)	7.16	6.75	0.20	21.6
Urea (mg/dL)	40.25	13.78	17	76
Creatinine (mg/dL)	1.22	0.24	0.5	1.7
INR	1.10	0.13	1	1.34
RBG (mg/dL)	114.0	31.09	86	230
HB (g/dL)	12.52	1.84	9.6	17.5
RBC (10^6^/mm^3^)	4.92	0.75	3.77	6.8
MCV (fL)	82.63	11.06	53	102
PLT (10^3^/mm^3^)	255.57	69.36	112	416
WBC (10^3^/mm^3^)	9.25	3.29	4.01	17.0
CEA (ng/dL)	14.56	18.54	2.1	76.0
CA19.9 (U/mL)	163.55	186.18	7.0	659

Site and size of pancreatic cancer

The most frequent site of pancreatic cancer was the head of the pancreas at 48% (n=24), followed by the head and neck at 26% (n=13), head and uncinate at 18% (n=9), and the body of the pancreas 8% (n=4). The size of pancreatic tumors measured by EUS (36.26 ± 14.38 mm) and CT (35.52 ± 14.47 mm) exhibited no significant difference, as shown in Table 4 and Table 5.

**Table 4:** Site of pancreatic cancer in the studied patients (n=50)

Site	Frequency (n) and the percentage(%)
Head	24 (48.0%)
Head neck	13 (26.0%)
Head and uncinate	9 (18.0%)
Body	4 (8.0%)
Total	50 (100.0%)

**Table 5:** Size of pancreatic cancer in the studied patients (n=50) CT, computed tomography; EUS, endoscopic ultrasound

	Size by CT	Size by EUS	P-value
Mean + SD	35.52 + 14.47 mm	36.26 + 14.38 mm	0.089
Median and range	35 (18-90) mm	35 (18-90) mm	

Histological type and TNM staging

Ductal adenocarcinoma accounted for 96% (n=48) of histological types observed among the studied patients. Adenosquamous carcinoma constituted 4% (n=2) of the cases. The comparison between CT and EUS staging indicated no significant difference in TNM staging, encompassing T, N, and M classifications and overall stage (IA, IB, IIA, IIB, III, IV) (see Table 6 and Table 7)

**Table 6:** Histological type of the studied patients

	Frequency (n)	Percentage (%)
Ductal adenocarcinoma	48	96%
Adenosquamous carcinoma	2	4%

**Table 7:** Stage of pancreatic cancer of studied patients (n=50) Data are presented as frequency (discrete numbers); t-test was used. CT, computed tomography; EUS, endoscopic ultrasound

TNM staging		By CT	By EUS	P-value
T	T1	7	5	0.083
	T2	24	25	
	T3	6	7	
	T4	13	13	
N	N0	13	13	0.99
	N1	22	22	
	N2	15	15	
M	M0	42	42	0.99
	M1	8	8	
IA		5	5	0.99
IB		7	7	
IIA		1	1	
IIB		21	21	
III		8	8	
IV		8	8	

Vascular invasion assessment

In the evaluation of vascular invasion, there was no significant difference between CT and EUS findings for the vessels assessed, which included the SMA, SMV, PV, hepatic artery (HA), CA, SPV, and SPA (Table 8).

**Table 8:** Vascular evaluation of the studied patients (n=50) SMA, superior mesenteric artery; SMV, superior mesenteric vein; PV, portal vein; HA, hepatic artery; CA, celiac artery; SPV, splenic vein; SPA, splenic artery; CT, computed tomography; EUS, endoscopic ultrasound

Vessel	CT			EUS			P-value
	No invasion (N)	Abutment (N)	Encasement (N)	No invasion (N)	Abutment (N)	Encasement (N)	
SMA	35	3	12	36	2	12	0.32
SMV	30	13	7	27	16	7	0.08
PV	45	4	1	43	6	1	0.16
HA	48	0	2	48	0	2	0.99
CA	48	0	2	48	0	2	0.99
SPV	48	0	2	48	0	2	0.99
SPA	46	0	4	46	0	4	0.99

Accuracy of EUS in vascular invasion

The diagnostic results of EUS for venous invasion evaluation demonstrated a sensitivity of 100%, specificity of 95.93%, PPV of 84.38%, NPV of 100%, and an accuracy of 96% compared to CT (Table 9).

**Table 9:** Accuracy of EUS in veins after correction of veins number CT, computed tomography; EUS, endoscopic ultrasound; NPV, negative predictive value; PPV, positive predictive value

EUS	CT			Diagnostic potential (%)	
	Venous invasion	No venous invasion	Total	Sensitivity	100% (27/27)
				Specificity	95.93% (118/123)
Venous invasion	27	5	32	PPV	84.38% (27/32)
No venous invasion	0	118	118	NPV	100% (118/118)
Total	27	123	150	Accuracy	96% (145/150)

Accuracy of EUS in arterial invasion

The diagnostic results of EUS for arterial invasion evaluation yielded a sensitivity of 95.65%, specificity of 100%, PPV of 100%, NPV of 99.44%, and an accuracy of 99.5% compared to CT (Table 10).

**Table 10:** Accuracy of EUS in arteries after correction of arterial number CT, computed tomography; EUS, endoscopic ultrasound

EUS	CT			Diagnostic potential (%)	
	Arterial invasion	No arterial invasion	Total	Sensitivity	95.65% (22/23)
				Specificity	100% (177/177)
Arterial invasion	22	0	22	PPV	100% (22/22)
No arteria invasion	1	177	178	NPV	99.44% (177/178)
Total	23	177	200	Accuracy	99.5% (199/200)

## Discussion

Pancreatic cancer stands as one of the most formidable malignancies in terms of prognosis, characterized by a discouraging five-year survival rate that scarcely exceeds 10% in the USA [10]. A small subset of patients is fortunate enough to be diagnosed with pancreatic cancer at an early stage, affording the possibility of radical surgical intervention. Nonetheless, even under such circumstances, the five-year survival rate struggles to surpass the 20% threshold [1]. The accurate evaluation of the intricate relationship between tumors and blood vessels is of paramount importance for determining the resectability of pancreatic cancer and predicting its prognosis. While CECT is currently considered the "gold standard" for the primary assessment of pancreatic cancer, the application of this technique faces limitations. Not all patients can tolerate CT evaluation involving intravenous contrast due to factors such as allergic reactions or impaired kidney function [4]. It is worth noting that approximately 0.6% of patients exhibit allergies to contrast medium. In this context, EUS is a valuable tool capable of rigorously evaluating pancreatic cancer and assessing vascular invasion without needing contrast agents [5-11].

Dahiya et al.'s nationwide survey in the USA from 2008 to 2017 highlighted a temporal gender shift in pancreatic cancer prevalence. While a slight female predominance was observed in 2008, a subsequent shift toward a slight male predominance was recorded in 2017 [12]. Furthermore, Cai et al. emphasized that pancreatic cancer is predominantly an affliction of the elderly, with a median age at diagnosis of 70 years. Interestingly, the percentage of individuals diagnosed before age 55 is quite limited [13]. The increasing number of hospitalizations among individuals aged 65-79 due to pancreatic cancer, as revealed by Dahiya et al., underscores the significance of age-related factors in the disease's epidemiology [12]. The notion of age-related telomere dysfunction, as explained by Matsuda, provides a potential rationale for the increased incidence of pancreatic cancer among the elderly [14].

Lugo et al.'s work offered insights into the relationship between smoking and pancreatic cancer risk, indicating a notable elevation in risk even with limited tobacco consumption. This observation reinforces our findings regarding the heightened risk associated with smoking [15]. The clinical presentation of pancreatic cancer in our study predominantly manifested as obstructive jaundice (66%), followed by abdominal pain (22%). Gohar et al.'s study yielded slightly different results, where abdominal pain was the dominant complaint (75%), followed by obstructive jaundice (21.1%) [11]. The divergences in clinical presentation may be attributed to the varying sample sizes between the studies.

Our study's observation of the head of the pancreas as the primary location for pancreatic cancer (92%) and the body as a less frequent site (8%) was corroborated by Gohar et al.'s findings [11]. However, Fujii et al. reported a distinct distribution, with 54.4% of cases in the head and 31.5% in the body [5]. Site distribution variability could stem from differing patient cohorts and geographic disparities. Moreover, while our study reported a tumor size of 35 (18-90) mm, Fujii et al. identified a smaller tumor size of 26 (18-33) mm [5]. The prevalence of ductal adenocarcinoma as the most common histological type in our study (94%) aligned with Fujii et al.'s findings (92.1%) [5].

Discrepancies between our study and Gohar et al.'s research in terms of tumor grading were evident, where the proportions of patients in different grading categories varied [11]. Regarding resectability, our study noted a resectable rate of 40%, whereas Gohar et al. recorded a resectable rate of 24% [11]. These disparities underscore the multifaceted nature of pancreatic cancer diagnosis and evaluation.

Our study demonstrated the diagnostic prowess of EUS in assessing venous and arterial invasion in pancreatic cancer. The high sensitivity, specificity, PPV, NPV, and accuracy observed in our study's EUS evaluations mirrored the results of Fujii et al. [5]. These findings highlight EUS as a reliable alternative to CT, particularly its ability to provide tissue samples and circumvent contrast agents.

Acknowledging the limitations of our study is crucial. These limitations encompassed a small sample size and the impact of utilizing the Al-Ibrashi Unit and Internal Medicine Hospital at Cairo University as an isolation facility during the COVID-19 pandemic, which inevitably affected case numbers. Additionally, we acknowledge the limitation that the study design is cross-sectional, which may impact the generalizability of our findings. Finally, the need for future studies to compare EUS and CT results with surgical findings in resectable cases is evident.

## Conclusions

Our study underscores the potential of EUS to accurately evaluate the staging and vascular invasion of pancreatic cancer. Its exceptional sensitivity, specificity, and accuracy suggest that EUS could surpass CT as the primary evaluation tool. Furthermore, EUS's capacity for tissue sampling and avoidance of potentially harmful contrast agents further reinforce its clinical value.
